# Vascular endothelial growth factor A polymorphisms and age-related macular degeneration: a systematic review and meta-analysis

**Published:** 2013-06-02

**Authors:** Chen Huang, Yongsheng Xu, Xuemin Li, Wei Wang

**Affiliations:** 1 Department of Ophthalmology, Peking University Third Hospital, Beijing, China; 2 Medical Research Center, Peking University Third Hospital, Beijing, China; 3 Clinical Stem Cell Center, Peking University Third Hospital, Beijing, China

## Abstract

**Purpose:**

In the present work, the aim was to systematically review all studies about the association of vascular endothelial growth factor A (*VEGF-A*) polymorphisms with age-related macular degeneration (AMD) and to perform a meta-analysis.

**Methods:**

Relevant studies were searched using PubMed, Embase, Wanfang (Chinese), VIP (Chinese), and the Chinese National Knowledge Infrastructure databases up to October, 2011. A meta-analysis was conducted using Stata software, version 11.0.

**Results:**

A total of nine studies with 2,281 AMD cases and 2,820 controls met our eligibility criteria, and meta-analyses of four polymorphisms of the *VEGF-A* gene (rs1413711, rs833061, rs2010963, and rs3025039) were performed. This meta-analysis revealed moderate evidence supporting an association between the *VEGF-A* polymorphisms and AMD. For rs1413711, the TT genotype was associated with an increased risk of overall AMD (TT versus CT model, odds ratio (OR) 1.74, 95% confidence interval (CI) 1.22–2.48) and of wet AMD (TT versus CT model, OR 1.82, 95% CI 1.22–2.71; TT versus (CC+CT) model, OR 1.63, 95% CI 1.13–2.35). For rs833061, the C allele (C allele versus T allele, OR 1.72, 95% CI 1.00–2.96) and CC genotype (CC versus TT model, OR 1.77, 95% CI 1.00–3.11) were the risk factors for overall AMD, while the C allele was also associated with an increased risk of wet AMD (C allele versus T allele, OR 1.54, 95% CI 1.03–2.31). No association was observed between AMD risk and the variant genotypes of *VEGF-A* rs2010963 and rs3025039 polymorphisms in different genetic models.

**Conclusions:**

The results suggest the *VEGF-A* rs1413711 and rs833061 polymorphisms may contribute to AMD susceptibility.

## Introduction

Age-related macular degeneration (AMD) is the most common cause of blindness, particularly irreversible blindness, in elderly people worldwide [1]. A recent review and meta-analysis of the prevalence of AMD in Asians reveals that the pooled prevalence estimates of early and late AMD in Asians aged 40–79 years old were 6.8% and 0.56%, respectively [2]. The findings of previous studies suggest AMD is a complex disease with demographic, environmental, and genetic risk factors [3]. The progression of AMD occurs over an extended time frame, with a primary influence on debris accumulation in the early stage and retinal pigment epithelial abnormalities in the late stage. There are two subtypes of late AMD, dry (atrophic) AMD and wet (neovascular) AMD [4], distinguishable by different clinical and pathologic features. The primary clinical characteristic of dry AMD is the appearance of retinal pigment epithelium (RPE) atrophy, usually known as geographic atrophy (GA).

Histopathologically, GA is usually the consequence of the loss of the RPE cell layer and overlying retinal photoreceptors, resulting in thinning of the retina and progressive visual impairment [3]. Wet AMD is the more devastating form and is mainly characterized by choroidal neovascularization (CNV) and subretinal neovascular fibrous tissue [3]. The new vessels invade the choroid and subretinal space, with subsequent exudation and bleeding. This results in scarring of the central retina and a loss of function [5].

Vascular endothelial growth factor (VEGF) is a key molecule in promoting angiogenesis and potentially inducing vascular leakage and inflammation by triggering the increased production and permeability of capillary endothelial cells [6]. The VEGF family includes placenta growth factor, VEGF-A, VEGF-B, VEGF-C, VEGF-D, and VEGF-E. Extensive evidence implies that increased VEGF-A expression plays a critical role in the CNV form of wet AMD. High concentrations of VEGF-A and its receptors are found in the CNV membrane, surrounding tissue, and RPE cells [7-9]. Recent therapies targeting VEGF significantly improve central vision, inhibit CNV, and delay the progression of AMD [10-12]. However, the clinical response to anti-VEGF therapy is inconsistent. Very little is known about why certain patients are treatment resistant. It is well known that the promoter region and introns within the *VEGF-A* gene may be important in regulating VEGF-A protein production and/or influencing mRNA splicing. Previous studies report that *VEGF-A* −460T/C and +634G/C were found to be associated with the risk of AMD in Caucasian AMD populations [13-15], and +936C/T was shown to significantly increase in wet AMD in a Chinese cohort [16]. Genetic variability of the *VEGF-A* gene may thus have an important role in determining and/or modifying the development and progression of AMD and the response to anti-VEGF therapy.

The *VEGF-A* gene is located on chromosome 6p21.3 and contains eight exons and seven introns [17]. Several single-nucleotide polymorphisms (SNPs) have been identified in the *VEGF-A* gene and are believed to have functional activity [18]. Among these, −460T/C (rs833061) in the promoter region, +405G/C (rs2010963) in the 5′-untranslated region, and +936C/T (rs3025039) in the 3′-untranslated region are known to modulate the protein expression of *VEGF-A* [18,19].

Over the past decade, a considerable number of epidemiological studies have focused on the association between *VEGF-A* polymorphisms and AMD susceptibility. However, these studies were limited by small or moderate sample sizes. Meta-analysis can be used to pool data from the appropriate individual studies to obtain sufﬁcient statistical power to detect the potential effect of small to moderate sizes of samples associated with these polymorphisms. To address these issues, we performed a systematic review and meta-analysis of all eligible case-control studies to estimate the association between VEGF-A polymorphisms and AMD risk.

## Methods

### Literature search

To identify the studies eligible for systematic review and meta-analysis, the following electronic databases were searched: PubMed, Embase, Wanfang (Chinese), VIP (Chinese), and the Chinese National Knowledge Infrastructure (CNKI), up to October 19, 2011. The following keywords were used: (VEGFA OR VEGF-A OR VEGF OR “vascular endothelial growth factor”) AND (haplotype OR polymorphism) AND (macular degeneration). The search was done without restrictions on language and included all studies conducted on human subjects. Additional studies were identified through a manual search of the references of the original studies. Of the studies with overlapping data published by the same investigators, only the most recent or complete study was included in this meta-analysis.

### Inclusion/exclusion criteria

Studies had to meet all of the following criteria: (i) they evaluated *VEGF-A* polymorphisms and AMD risk, (ii) they were case-control or cohort studies, and (iii) they contained sufficient published data to estimate an odds ratio (OR) with a 95% confidence interval (CI). Following the application of these criteria, meta-analyses were performed for all polymorphisms for which eligible data were reported in at least three published studies.

### Data extraction

Information was carefully extracted by two independent investigators according to the inclusion criteria noted above. For each study, the following information was collected: the first author’s surname, year of publication, country of origin, ethnicity, mean age and type of cases and controls, and the number of cases and controls for each genotype of *VEGF-A* polymorphisms. Ethnic origins were categorized as Caucasian, Asian, and African. If a study did not state the ethnic descendent or if it was not possible to separate participants according to such phenotypes, the group was termed “mixed.” Late AMD was divided into two subtypes: dry AMD (nonexudative AMD, atrophic AMD, or geographic atrophy) and wet AMD (exudative AMD, neovascular AMD, or choroidal neovascularization).

### Statistical analysis

To test for control population selective bias, a chi-square test was applied to determine if the genotype distribution of the control subjects of each individual population conformed to the Hardy-Weinberg equilibrium (HWE; p<0.05 was considered significant). Associations between *VEGF-A* polymorphisms and AMD risk were calculated using ORs and 95% CIs. The genetic model analysis was performed according to the Thakkinstian et al. [20] method, and then the wild-type allele was set as A and the risk allele as B. For each polymorphism, A and B allele frequencies were first compared in case and control groups. The best genetic model was determined by estimating the three possible ORs and their 95% CI in the meta-analysis sample: OR1 (BB versus AA), OR2 (AB versus AA), and OR3 (BB versus AB). If OR1=OR3≠1 and OR2=1, then a recessive model (BB versus AA+AB) is suggested. If OR1=OR2≠1 and OR3=1, a dominant model (BB+AB versus AA) is suggested. If OR1=OR2=1 and OR3 ≠1, a complete overdominant model (AA+BB versus AB) is suggested. If OR1>OR2>1 and OR1>OR3>1 (or OR1<OR2<1 and OR1<OR3<1), a co-dominant model (AB versus AA and BB versus AA) is suggested. When none of the OR values significantly deviated from 1, meta-analyses were performed for these different genetic models. Sensitivity analysis was used to examine the effect of excluding specific studies, such as studies with controls that were not in HWE. The statistical significance of the summary OR was determined using the *Z* test, in which p<0.05 was considered significant. Between-study heterogeneity was estimated using the χ^2^-based Q statistic [21]. Heterogeneity was considered statistically significant when p<0.1 or I^2^>50% [22]. If heterogeneity existed, data was analyzed using a random effects model. In the absence of heterogeneity, a fixed effects model was used. The Begg’s rank correlation method and the Egger’s weighted regression method were used to assess potential publication bias. All statistical analyses were performed using Stata software (version 11.0; Stata Corporation, College Station, TX) and two-sided *p* values.

## Results

**Figure 1:**
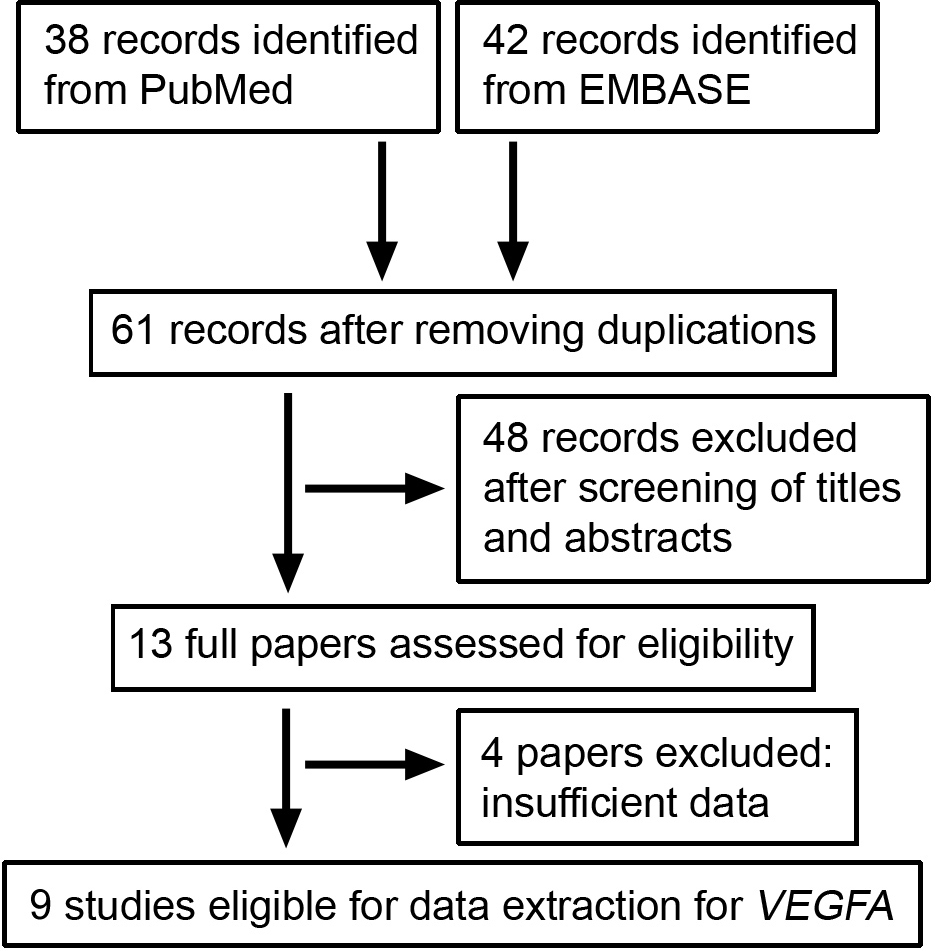
Flow for identifying and selecting studies in this meta-analysis.

Eighty potentially relevant papers were retrieved (38 in PubMed, 42 in Embase, 0 in Wanfang, 0 in VIP, and 0 in CNKI). Sixty-seven studies were subjected to a full-text review and excluded according to the selection criteria stated above. Thirteen studies were identified that examined the association between *VEGF-A* polymorphisms and AMD risk [13-16,23-31] and four of these studies were excluded for insufficient data [14,29-31]. As summarized in Table 1 and Figure 1, data were available from a total of nine studies [13,15,16,23-28], with 2,281 AMD patients and 2,820 control subjects in total. The definitions of AMD patients and controls in these studies were based on clinical ophthalmic examinations and various grading systems, such as the Clinical Age-Related Maculopathy Staging (CARMS) system [24], the International Age-Related Maculopathy (ARM) Epidemiologic Study [16,26], the Age-Related Eye Disease Study (AREDS) [27], and others [13,15,23,25,28] (Appendix 1). Controls were defined as those subjects having no clinical evidence of AMD, without detectable drusen or pigmentary abnormalities, or without drusen of more than 63 μm in both eyes.

**Table 1:** Characteristics of eligible studies included in this study

**Reference**	**Country (ethnicity)**	**Cases/ Controls**	**Genotyping method**	**Source of controls**	**Type of controls**	**Type of cases**	**Polymorphisms of VEGF-A gene**
[23]	China (Asian)	159/140	MassArray	Hospital	Age, gender, and ethnicity matched without AMD	Neovascular AMD	rs833061, rs1413711, rs2010963, rs3025039
[24]	Brazil (mixed)	160/140	TaqMan	Hospital	Age and gender matched without AMD,	Exudative and nonexudative AMD	rs1413711
[25]	Finland (Caucasian)	162/85	TaqMan	Hospital	Age matched without AMD	Exudative AMD	rs699947, rs2146323, rs3025033
[26]	Italy (Caucasian)	226/248	PCR-SBE	Hospital	Gender and ethnicity matched without detectable drusen	Neovascular and nonneovascular AMD	rs833068, rs833069, rs3024994, rs3025007, rs3025039
[15]	Poland (Caucasian)	265/136	Allele-specific PCR and PCR-RFLP	Hospital	Age and gender matched without AMD	Atrophic and neovascular AMD	rs833061, rs2010963
[27]	USA (Caucasian)	211/187		Hospital	Without AMD	Atrophic and neovascular AMD	rs833070
[16]	China (Asian)	190/180	PCR-RFLP	Hospital	Age and gender matched without any type of drusen, geographic atrophy, CNV, or other retinal disorder in either eye	Atrophic and neovascular AMD	rs699947, rs833061, rs1413711, rs2010963, rs3025039
[28]	Australia (Caucasian)	577/173	MassArray	Population	Ethnicity and residence matched without AMD	Early, atrophic, and neovascular AMD	rs10434, rs25648, rs833061, rs2146323, rs3024997, rs3025030, rs3025035
[13]	UK (Caucasian)	45/94	PCR	Hospital	Age matched healthy	Neovascular AMD	rs735286, rs1413711, rs2146323, rs3025021, rs3025024

*PCR* polymerase chain reaction; *SBE* single base extension; *RFLP* restriction fragment length polymorphism.

**Figure 2:**
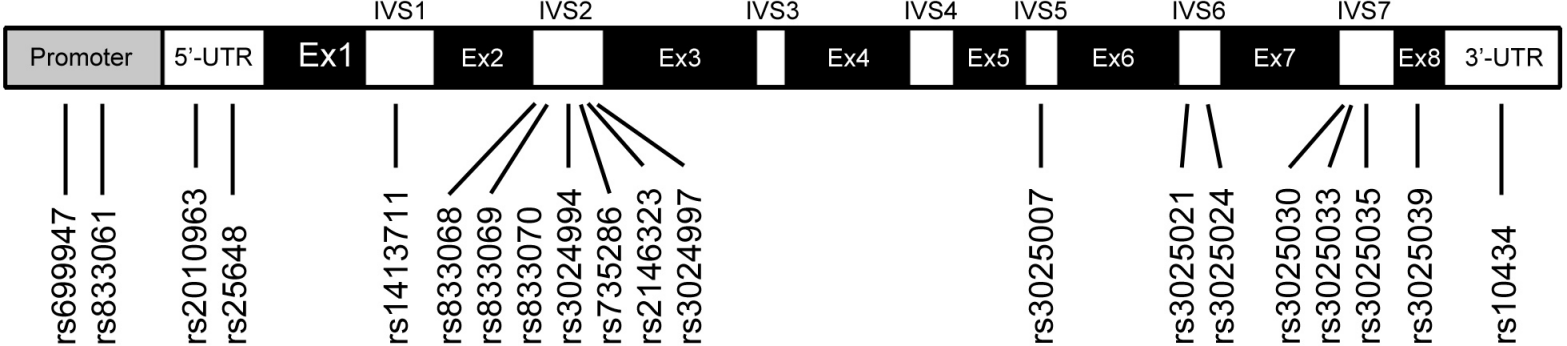
The *VEGF-A* polymorphisms used in eligible studies. Ex, exon; IVS, intervening sequence.

These studies focused on 20 identified polymorphisms of the *VEGF-A* gene (Figure 2): rs699947 and rs833061 in the promoter region, rs2010963 and rs25648 in the 5′ UTR, rs1413711 in the intron 1, rs833068, rs833069, rs833070, rs3024994, rs735286, rs2146323, and rs3024997 in the intron 2, rs3025007 in the intron 5, rs3025021 and rs3025024 in the intron 6, rs3025030, rs3025033, and rs3025035 in the intron 7, rs3025039 in the exon 8, and rs10434 in the 3′ UTR. Data reported in at least three published studies were available for four *VEGF-A* polymorphisms (rs1413711, rs833061, rs2010963, and rs3025039). The lists of genotypes and allelic frequencies of these four *VEGF-A* polymorphisms in the eligible studies are provided in Table 2. A study [16] investigating the rs2010963 polymorphism significantly deviated from HWE (p<0.05).

**Table 2:** The genotype distribution of *VEGF-A* polymorphisms used in this study

**Polymorphism**	**Reference**	**Ethnicity**	**Type of cases**	**Cases**	**Controls**	**HWE**
rs1413711			**CC/CT/TT**	**CC/CT/TT**		
	[23]	Asian	wet AMD	81/58/20	81/50/9	0.73
	[24]	Mixed	total AMD	65/66/29	67/65/8	0.12
			dry AMD	14/14/8		
			wet AMD	51/52/21		
	[16]	Asian	total AMD	57/80/53	50/85/42	0.61
			dry AMD	29/46/29		
			wet AMD	28/34/24		
	[13]	Caucasian	wet AMD	17/18/10	19/54/21	0.14
rs833061				**TT/TC/CC**	**TT/TC/CC**	
	[23]	Asian	wet AMD	81/58/20	81/50/9	0.73
	[15]	Caucasian	total AMD	48/191/26	60/63/11	0.32
			dry AMD	13/67/8		
			wet AMD	35/124/18		
	[16]	Asian	total AMD	116/66/8	116/60/4	0.23
			dry AMD	60/38/6		
			wet AMD	56/28/2		
	[28]	Caucasian	total AMD	154/259/153	40/78/39	0.93
			dry AMD	27/43/30		
			wet AMD	92/158/86		
rs2010963			**GG/GC/CC**	**GG/GC/CC**		
	[23]	Asian	wet AMD	54/70/35	39/74/27	0.44
	[15]	Caucasian	total AMD	164/84/17	85/44/5	0.81
			dry AMD	47/30/11		
			wet AMD	117/54/6		
	[16]	Asian	total AMD	40/132/18	34/116/30	0
			dry AMD	24/70/10		
			wet AMD	16/62/8		
rs3025039			**CC/CT/TT**	**CC/CT/TT**		
	[23]	Asian	wet AMD	114/33/12	92/40/8	0.2
	[26]	Caucasian	total AMD	175/48/3	190/54/4	0.94
	[16]	Asian	total AMD	120/58/12	134/42/4	0.74
			dry AMD	75/27/2		
			wet AMD	45/31/10		

### rs1413711

The rs1413711 SNP (+674C/T) is located in intron 1 of the *VEGF-A* gene (1695 bp downstream from the start of exon 1) and has been investigated in association studies in patients with AMD, particularly wet AMD. A positive association was initially reported by Churchill et al. [13], with an excess frequency of the CC genotype in a Caucasian sample of wet AMD patients versus controls. However, this observation was not replicated in subsequent studies. Lin et al. [16] studied a Chinese population and reported no association between the rs1413711 polymorphism of the *VEGF-A* gene and AMD. More recently, Almeida et al. [24] and Qu et al. [23] reported significant associations in Brazilian and Chinese populations between AMD and the TT genotype as the risk genotype.

**Figure 3:**
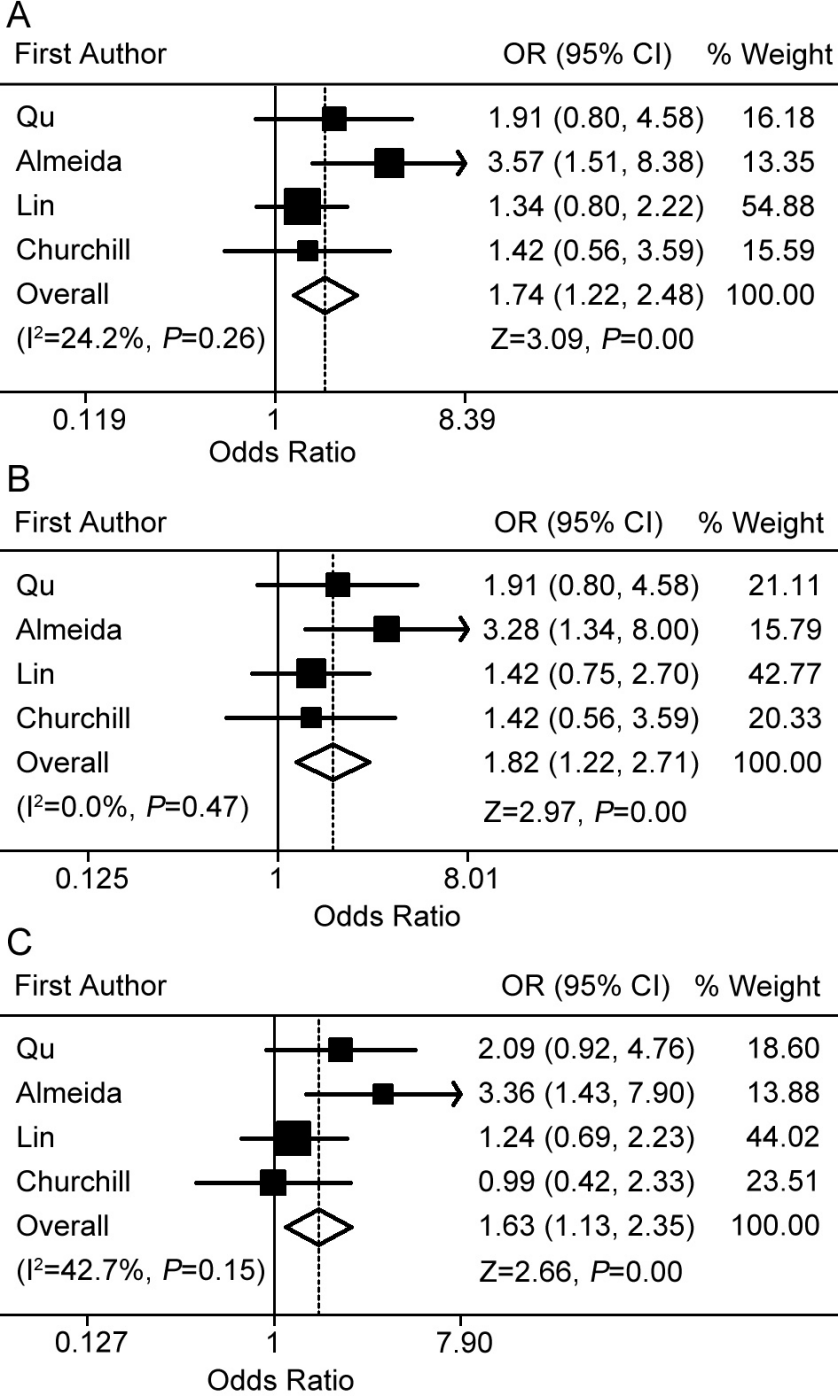
Forest plots for the association between the *VEGF-A* rs1413711 allele and AMD risk. **A**: Results from fixed effects meta-analysis of TT versus CT model in the overall comparison. **B**: Results from fixed effects meta-analysis of TT versus CT model in the stratified analysis for wet AMD. **C**: Results from fixed effects meta-analysis of TT versus CC+CT model in the stratified analysis for wet AMD.

In this meta-analysis, the four studies [13,16,23,24] with the rs1413711 polymorphism of the *VEGF-A* gene comprised three different populations, including 554 AMD cases and 551 controls. An evaluation of the association between the *VEGF-A* rs1413711 polymorphism and AMD risk is presented in Table 3. Considering all individuals from those studies, no significant association between AMD and the T allele was detected (random effects OR 1.15 95% CI 0.86–1.56). As shown in Figure 3A, the *VEGF-A* rs1413711 TT genotype was associated with a significantly increased AMD risk in the TT versus the CT model (fixed effects OR 1.74, 95% CI 1.22–2.48). No significant results were observed in any other genetic models. In the stratified analysis for wet AMD, the *VEGF-A* rs1413711 TT genotype was also associated with a significantly increased wet AMD risk in the TT versus the CT model (fixed effects OR 1.82, 95% CI 1.22–2.71) and the TT versus the CC+CT model (fixed effects OR 1.63, 95% CI 1.13–2.35; Table 3 and Figure 3).

**Table 3:** Results of meta-analysis of the association between rs1413711 polymorphism of *VEGF-A* gene and AMD risk

**Genotype contrast**	**N^a^**	**Cases/Controls**	**OR (95% CI)**	**Significance (Z test)^b^**		**Heterogeneity(Q test)**		
				**Z**	**P**	**Q**	**I^2^ (%)**	**P**
**overall**	**4**	**554/551**						
T versus C			1.15 (0.86–1.56)	0.98	0.32	7.96	62.3	0.04
TT versus CC			1.50 (0.71–3.16)	1.07	0.28	10.51	71.5	0.01
CT versus CC			0.90 (0.69–1.18)	0.72	0.47	5.72	47.6	0.12
TT versus CT			1.74 (1.22–2.48)	3.09	0	3.96	24.2	0.26
(CT+TT) versus CC			0.98 (0.65–1.49)	0.05	0.95	7.72	61.1	0.05
TT versus (CC+CT)			1.69 (0.98–2.89)	1.92	0.05	6.63	54.8	0.08
(CC+TT) versus CT			1.23 (0.97–1.57)	1.73	0.08	2.84	0	0.41
**wet AMD**	**4**	**414/551**						
T versus C			1.13 (0.83–1.54)	0.8	0.42	7.32	59	0.06
TT versus CC			1.44 (0.68–3.05)	0.95	0.34	9.5	68.4	0.02
CT versus CC			0.82 (0.54–1.26)	0.87	0.38	6.12	51	0.1
TT versus CT			1.82 (1.22–2.71)	2.97	0	2.51	0	0.47
(CT+TT) versus CC			0.94 (0.60–1.48)	0.24	0.81	7.86	61.8	0.04
TT versus (CC+CT)			1.63 (1.13–2.35)	2.66	0	5.24	42.7	0.15
(CC+TT) versus CT			1.25 (0.96–1.63)	1.69	0.09	3.07	2.4	0.38

^a^Number of studies. ^b^Random-effects model was used when *p* value for heterogeneity test p<0.1 or I^2^>50%; otherwise, fixed-effects model was used.

### rs833061

The rs833061 SNP (−460 T/C) is located in the promoter region of the *VEGF-A* gene. A pioneer study by Richardson et al. [28] of a Caucasian population showed the rs833061 polymorphism of the *VEGF-A* gene was not associated with dry or wet AMD. Furthermore, two studies [16,23] of a sample Chinese AMD population reported no association. Janik-Papis et al. [15] reported a positive association in a Polish population. The TC genotype was associated with a significantly increased risk of AMD, including dry AMD and wet AMD.

**Figure 4:**
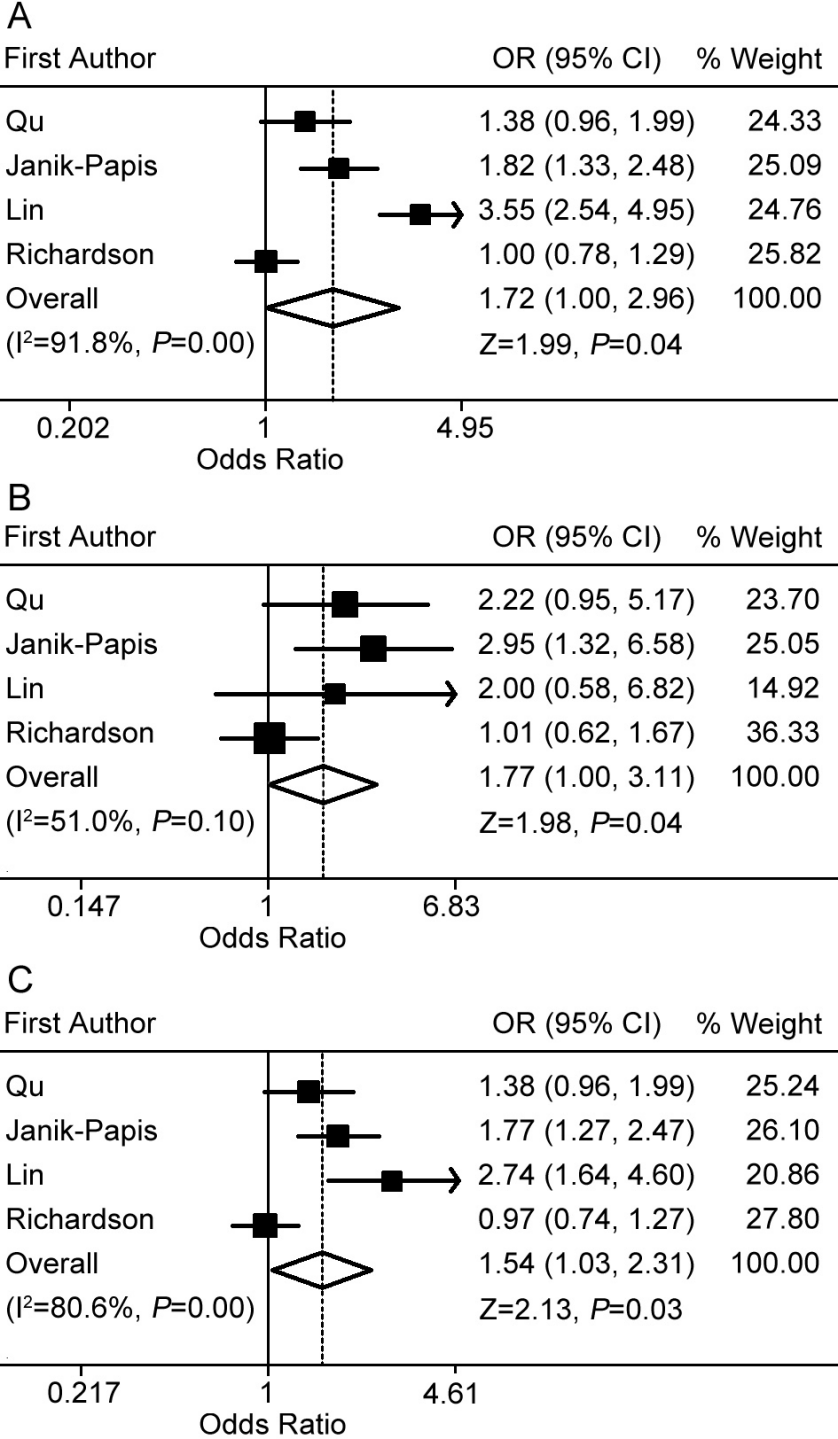
Forest plots for the association between the *VEGF-A* rs833061 allele and AMD risk. **A**: Results from random effects meta-analysis of C allele versus T allele in the overall comparison. **B**: Results from random effects meta-analysis of CC versus TT model in the overall comparison. **C**: Results from random effects meta-analysis of C allele versus T allele model in the stratified analysis for wet AMD.

We retrieved four studies comprising four different sample populations (two Caucasian and two Asian) for whom detailed allele frequencies were reported, including 1,180 cases and 611 controls. Overall, significant associations were found for the C allele versus the T allele (random effects OR 1.72, 95% CI 1.00–2.96), and for the CC versus the TT model (random effects OR 1.77, 95% CI 1.00–3.11; Table 4 and Figure 4). In the stratified analysis for dry AMD, no significant results were observed in any of the genetic models. A significantly elevated risk was associated with the *VEGF-A* rs833061 C allele among subjects with wet AMD (C allele versus T allele: random effects OR 1.54, 95% CI 1.03–2.31; Table 4 and Figure 4C).

**Table 4:** Results of meta-analysis of the association between rs833061 polymorphism of *VEGF-A* gene and AMD risk.

**Genotype contrast**	**N^a^**	**Cases/ Controls**	**OR (95% CI)**	**Significance (Z test)^b^**		**Heterogeneity (Q test)**		
				**Z**	**P**	**Q**	**I^2^ (%)**	**P**
**overall**	**4**	**1180/611**						
C versus T			1.72 (1.00–2.96)	1.99	0.04	36.44	91.8	0
CC versus TT			1.77 (1.00–3.11)	1.98	0.04	6.12	51	0.1
TC versus TT			1.42 (0.75–2.69)	1.08	0.27	23.53	87.3	0
CC versus TC			1.21 (0.87–1.69)	1.15	0.24	2.77	0	0.42
(TC+CC) versus TT			1.49 (0.83–2.68)	1.35	0.17	21.72	86.2	0
CC versus (TT+TC)			1.30 (0.94–1.78)	1.63	0.1	2.24	0	0.52
(TT+CC) versus TC			0.78 (0.45–1.34)	0.89	0.37	20.23	85.2	0
**dry AMD**	**3**	**292/471**						
C versus T			1.99 (0.94–4.18)	1.82	0.06	24.88	92	0
CC versus TT			1.68 (0.99–2.84)	1.93	0.05	3.47	42.3	0.17
TC versus TT			1.67 (0.63–4.43)	1.03	0.3	15.73	87.3	0
CC versus TC			1.25 (0.78–2.01)	0.93	0.35	2.49	19.7	0.28
(TC+CC) versus TT			1.75 (0.73–4.19)	1.26	0.2	13.73	85.4	0
CC versus (TT+TC)			1.37 (0.87–2.15)	1.38	0.16	1.27	0	0.53
(TT+CC) versus TC			0.69 (0.29–1.63)	0.84	0.39	15.65	87.2	0
**wet AMD**	**4**	**758/611**						
C versus T			1.54 (1.03–2.31)	2.13	0.03	15.43	80.6	0
CC versus TT			1.44 (0.98–2.10)	1.88	0.06	5.73	47.7	0.12
TC versus TT			1.34 (0.73–2.45)	0.97	0.33	17.21	82.6	0
CC versus TC			1.14 (0.79–1.63)	0.73	0.46	1.99	0	0.57
(TC+CC) versus TT			1.39 (0.79–2.45)	1.15	0.25	16.77	82.1	0
CC versus (TT+TC)			1.22 (0.87–1.71)	1.18	0.23	2.21	0	0.53
(TT+CC) versus TC			0.80 (0.48–1.33)	0.84	0.4	20.23	78.7	0

^a^Number of studies. ^b^Random-effects model was used when P value for heterogeneity test p<0.1 or I^2^>50%; otherwise, fixed-effects model was used.

### rs2010963

The rs2010963 allele (+405C/G or −634G/C) is located in the 5′-untranslated region of the *VEGF-A* gene. Studies by Lin et al. [16] and Qu et al. [23] of two Chinese populations reported no association between the rs2010963 polymorphism of the *VEGF-A* gene and AMD. Janik-Papis et al. reported positive associations of the C allele, CC genotype, and the occurrence of dry AMD [15].

A meta-analysis of an association in three studies of AMD patients (614 cases and 454 controls from one Caucasian population and two Asian populations) did not show a significant association in any of the genetic models of the overall comparison or in the stratified analysis for wet AMD (Table 5). A sensitivity analysis was performed after excluding the study conducted by Lin et al [16] because the controls were not in HWE; however, this did not alter the pattern of results (data not shown).

**Table 5:** Results of meta-analysis of the association between rs2010963 polymorphism of *VEGF-A* gene and AMD risk.

**Genotype contrast**	**N^a^**	**Cases/ Controls**	**OR (95% CI)**	**Significance (Z test)^b^**		**Heterogeneity (Q test)**		
				**Z**	**P**	**Q**	**I^2^ (%)**	**P**
**overall**	**3**	**614/454**						
C versus G			0.93 (0.77–1.12)	0.7	0.48	1.77	0	0.41
CC versus GG			0.86 (0.56–1.32)	0.69	0.49	3.83	47.8	0.14
GC versus GG			0.88 (0.66–1.17)	0.87	0.38	1.28	0	0.52
GC versus CC			1.02 (0.49–2.15)	0.07	0.94	6.07	67.1	0.04
(GC+CC) versus GG			0.90 (0.68–1.18)	0.72	0.47	1.14	0	0.56
CC versus (GG+CG)			0.96 (0.49–1.89)	0.1	0.91	5.48	63.5	0.06
(GG+CC) versus GC			1.05 (0.81–1.35)	0.38	0.7	3.28	39.1	0.19
**wet AMD**	**3**	**422/454**						
C versus G			0.90 (0.73–1.11)	0.94	0.34	0.09	0	0.95
CC versus GG			0.81 (0.49–1.32)	0.83	0.4	0.71	0	0.69
GC versus GG			0.85 (0.62–1.17)	0.98	0.32	1.42	0	0.49
GC versus CC			0.95 (0.61–1.48)	0.22	0.82	3.7	46	0.15
(GC+CC) versus GG			0.85 (0.63–1.16)	0.99	0.32	0.56	0	0.75
CC versus (GG+CG)			0.88 (0.58–1.35)	0.54	0.58	2.68	25.4	0.26
(GG+CC) versus GC			1.08 (0.81–1.43)	0.56	0.57	3.7	46	0.15

^a^Number of studies. ^b^Random-effects model was used when P value for heterogeneity test p<0.1 or I^2^>50%; otherwise, fixed-effects model was used.

### rs3025039

The rs3025039 allele (+936C/T) is located in the 3′-untranslated region of the *VEGF-A* gene. The three studies [16,23,26] with the rs3025039 polymorphism of the *VEGF-A* gene comprised three different populations, including 575 AMD cases and 568 controls from one Caucasian population and two Asian populations. Lin et al. [16] reported that the T allele of the rs3025039 SNP was significantly increased in Chinese wet AMD patients compared with controls. However, this association was not replicated in the other groups, i.e., in one Caucasian population [26] and one Chinese population [23].

In an overall comparison of three studies, there was no association between this polymorphism and the risk of AMD in any of the genetic models (Table 6). Due to the limited data, we did not analyze the data based on the subtype of AMD for this polymorphism.

**Table 6:** Results of meta-analysis of the association between rs3025039 polymorphism of *VEGF-A* gene and AMD risk.

**Genotype contrast**	**N^a^**	**Cases/ Controls**	**OR (95% CI)**	**Significance (Z test)^b^**		**Heterogeneity (Q test)**		
				**Z**	**P**	**Q**	**I^2^ (%)**	**P**
**overall**	**3**	**575/568**						
T versus C			1.12 (0.74–1.70)	0.56	0.57	6.66	70	0.03
TT versus CC			1.60 (0.85–3.03)	1.46	0.14	2.68	25.3	0.26
CT versus CC			1.01 (0.63–1.59)	0.04	0.96	5.49	63.6	0.06
CT versus TT			1.66 (0.84–3.28)	1.47	0.14	0.96	0	0.61
(CT+TT) versus CC			1.07 (0.67–1.71)	0.31	0.75	6.3	68.2	0.04
TT versus (CC+CT)			1.62 (0.86–3.05)	1.5	0.13	2	0	0.36
(CC+TT) versus CT			1.01 (0.66–1.55)	0.07	0.94	4.88	59	0.08

^a^Number of studies. ^b^Random-effects model was used when P value for heterogeneity test p<0.1 or I^2^>50%; otherwise, fixed-effects model was used.

### Publication bias

The Begg’s rank correlation method and Egger’s weighted regression method were used to assess publication bias. No obvious publication bias for these polymorphisms was found.

## Discussion

The VEGF-A protein is the most important regulator of angiogenesis and is overexpressed in the retinal tissue of AMD, particularly wet AMD [7-9]. Polymorphisms that can alter VEGF expression and protein production may contribute to the risk of AMD. Several published studies have been conducted in recent years to evaluate the association between the *VEGF-A* SNPs in terms of AMD risk predisposition in different ethnic populations, but the results have been conflicting [13-16,23-31]. Churchill et al. [13] found the rs1413711 CC genotype was significantly associated with an increased susceptibility to AMD in a Caucasian population. Richardson et al. [28] investigated seven tSNPs of the *VEGF-A* gene and found no evidence of an association between these SNPs and either AMD or any of its subtypes in an Australian population. Lin et al. [16] and Qu et al. [23] analyzed the association of SNPs in the *VEGF-A* gene with the risk of AMD in Chinese patients. Lin et al. [16] suggested that the rs3025039 allele was significantly associated with wet AMD. However, Qu et al. [23] reported that no evident association was found in the allele frequencies of any individual SNP between AMD patients and controls.

In the present study, a systematic review/meta-analysis was performed to examine the association between the *VEGF-A* polymorphisms and AMD risk by critically reviewing nine studies, including a total of 2,281 AMD patients and 2,820 controls. These studies focused on 20 identified polymorphisms of the *VEGF-A* gene, but only four polymorphisms (rs1413711, rs833061, rs2010963, and rs3025039) were reported in at least three published studies.

The rs1413711 polymorphism, also called +674C/T, is located in intron 1 of the *VEGF-A* gene. Thus far, no data are available regarding the functional activity of this polymorphism. Our meta-analysis on the available studies showed the *VEGF-A* rs1413711 TT genotype was associated with a significantly increased AMD risk. People who had the rs1413711 TT genotype had a 74 percent higher risk of AMD than people who had the CT genotype. In the stratified analysis by subtypes of AMD, it was also found that the rs1413711 TT genotype was a risk factor in wet AMD patients. The TT genotype carriers had a nearly 82 percent increased risk of wet AMD compared with the CT genotype carriers, and had about a 63 percent increased risk of wet AMD in the recessive model (TT versus CC+CT). Previous studies have suggested VEGF is a pathogenic factor in the development of CNV [7-9]. Churchill et al. [13] considered that proximity of this SNP may alter the local environment, influence binding, and increase VEGF production. Further studies are needed to clarify the biologic function of the rs1413711 polymorphism and the influence of the TT genotype on the pathogenesis and clinical treatment of AMD, particularly wet AMD.

The rs833061 allele (−460 T/C) is located in the promoter region of the *VEGF-A* gene and the T allele may be associated with decreased promoter activity of the *VEGF-A* gene [19]. In the present meta-analysis, the C allele and the CC genotype were associated with a significantly increased AMD risk. The individuals with the rs833061 C allele had a 72 percent higher risk of AMD compared with individuals with the rs833061 T allele, and those with the CC genotype had a 77 percent higher risk of AMD compared with those with the TT genotype. In the stratified analysis for wet AMD, the C allele carriers had about a 54 percent increased risk of wet AMD compared with the T allele carriers, demonstrating a significant effect of the C allele on the increased risk for wet AMD. However, in the stratified analysis for dry AMD, no significant associations were observed in any of the genetic models. This suggests a potential difference in etiologic mechanisms and the clinical response of anti-VEGF therapy between dry AMD and wet AMD. Agents that block the effects of VEGF are emerging as the most successful treatment for wet AMD [32-34]. The carriage of the C allele and the CC genotype of the rs833061 polymorphism is potentially a risk factor for AMD, and the association between this SNP genotype and the clinical response of anti-VEGF treatment requires further study. Moreover, the sample size, especially of dry AMD, in the present study was still limited and potential small genetic effects may not be detectable. Such findings warrant further studies with larger samples and more homogenous populations.

In this meta-analysis, it was found that the *VEGF-A* rs2010963 and rs3025039 polymorphisms were not a risk factor for AMD. When stratifying for the subtype of the disease, there were no significant differences in genotype distribution between wet AMD cases and controls.

It should be noted that there were some limitations in this study. First, because of the limitations of raw data and publication, some relevant studies were excluded from this meta-analysis. Second, the sample sizes in the analyses were extremely small. Third, the sources of heterogeneity that existed among studies for most polymorphisms were not addressed. Finally, this meta-analysis was based on unadjusted data, while a more precise analysis could be performed if individual data were available.

In conclusion, despite the above-mentioned limitations, this meta-analysis suggests the TT genotype of rs1413711 and the C allele and the CC genotype of rs833061 in the *VEGF-A* gene are associated with an increased risk of overall AMD, and the TT genotype of rs1413711 and the C allele of rs833061 are a risk factor in wet AMD. *VEGF-A* rs2010963 and rs3025039 polymorphisms show no association with AMD risk. More detailed and well designed studies with larger populations and different ethnicities are needed to further evaluate the associations.

## Appendix 1. Definition of cases with age-related macular degeneration and controls included in this study.

To access the data, click or select the words “Appendix 1.” This will initiate the download of a compressed (pdf) archive that contains the file. OCT optical coherence tomography; FA fluorescein angiography; ARM age-related maculopathy; GA geographic atrophy; CNVM choroidal neovascular membrane.
